# Clinical value of CA125, AFP, and CEA for combined diagnosis and assessment of gastric cancer prognosis

**DOI:** 10.3389/fonc.2025.1530522

**Published:** 2025-05-08

**Authors:** Yanhai Jia, Yajuan Wang, Haiying Li, Yamei Yang, Min Sun

**Affiliations:** Laboratory Department, Baoji People's Hospital, Baoji, Shaanxi, China

**Keywords:** CA125, AFP, CEA, gastric cancer, pathological feature, clinical prognosis, application value

## Abstract

To analyze the diagnostic and prognostic value of combined detection of CA125, AFP, and CEA for gastric cancer. Ninety-eight gastric cancer patients treated in our hospital from January 2020 to November 2022 were retrospectively selected and classified into the gastric cancer group according to screening criteria, while 80 patients diagnosed with benign gastric lesions during the same period were classified into the benign group. Serum levels of CA125, AFP, CEA, and their positive rates were significantly higher in the gastric cancer group compared to the benign group (P<0.05). The AUCs for CA125, AFP, CEA, and their combined detection in diagnosing gastric cancer were 0.815, 0.813, 0.911, and 0.919, respectively (P<0.001). In patients with stage III-IV, the levels of CA125, AFP, and CEA were higher than those in stage I-II (P<0.05). The AUCs for serum CA125, AFP, CEA, and their combined detection in TNM staging of gastric cancer were 0.751, 0.834, 0.911, and 0.931, respectively (P<0.001). Poorly differentiated patients had higher levels of CA125, AFP, and CEA compared to moderately to well-differentiated patients (P<0.05). The AUCs for serum CA125, AFP, CEA, and their combined detection in diagnosing differentiation degree were 0.819, 0.883, 0.746, and 0.986, respectively (P<0.001). Patients with metastasis had higher levels of CA125, AFP, and CEA compared to those without metastasis (P<0.05). The AUCs for serum CA125, AFP, CEA, and their combined detection in diagnosing metastasis were 0.716, 0.825, 0.863, and 0.892, respectively (P<0.001). The levels of CA125, AFP, and CEA of patients in the death group were higher than those in the survival group (P<0.05). The AUCs for serum CA125, AFP, CEA, and their combined detection in predicting clinical outcomes of gastric cancer patients were 0.713, 0.809, 0.922, and 0.926, respectively (P<0.001). Cox regression analysis indicated that TNM staging, peritoneal metastasis, and elevated CEA levels were independent risk factors for poor prognosis (mortality) in patients with gastric cancer (P<0.05). Serum levels of CA125, AFP, and CEA in patients with gastric cancer were significantly elevated and were correlated with the degree of differentiation and TNM staging. Combined detection had diagnostic efficacy in assessing metastasis and clinical outcomes.

## Introduction

Gastric cancer is among the most prevalent malignancies worldwide, ranking fourth in incidence and second in mortality among malignant tumors globally (1, 2). In several Asian countries such as Japan, South Korea, and China, gastric cancer remains highly prevalent. Despite a global decline in its incidence in recent years, gastric cancer continues to be one of the major malignant tumors threatening the health of the Chinese people, and the diagnosis, prevention, and treatment of gastric cancer remain critical tasks for medical professionals in China (3).

At present, surgery remains the preferred treatment for gastric cancer. Accurate tumor staging and diagnosis directly impact the implementation of surgical intervention and are closely associated with the prognosis of patients (4). Currently, the diagnosis and staging assessment of gastric cancer still rely on pathological examinations as the gold standard. Although imaging tests can be used for diagnosis and staging of gastric cancer, they have certain limitations, such as difficulty in distinguishing whether enlarged lymph nodes are due to inflammation or tumor metastasis (5).

Serological markers have been extensively studied for their application in assessing malignant tumor conditions, diagnosing diseases, and evaluating prognoses. Compared to pathological examinations and imaging tests, serological tests have the advantages of strong reproducibility, low cost, and convenient sampling (6). Research (7) indicates that malignant tumors secrete substances with specific biological activities in the form of enzymes, proteins, and hormones, due to abnormal gene expression, and these levels are often closely associated with the onset and progression of tumors.

Carbohydrate antigen 125 (CA125) is a high-molecular-weight glycoprotein initially regarded as a specific biomarker for ovarian cancer. However, recent studies have demonstrated that CA125 is also highly expressed in various digestive system tumors, including gastric and pancreatic cancers. CA125 is primarily secreted by mesothelial and epithelial tumor cells. In the malignant tumor microenvironment, CA125 not only involves in tumor cell proliferation but also facilitates immune evasion by suppressing natural killer cell activity, thereby accelerating tumor progression (8). Elevated serum CA125 levels in gastric cancer patients often indicate advanced disease or peritoneal metastasis. Existing research has established a negative correlation between CA125 levels and overall survival in gastric cancer patients (9). Alpha-fetoprotein (AFP) is a glycoprotein synthesized by the yolk sac and fetal liver during embryonic development, with minimal expression in normal adult serum. However, its levels are elevated in primary hepatocellular carcinoma, germ cell tumors, and certain gastric cancers. Elevated AFP in gastric cancer may be associated with tumor cells exhibiting hepatoid differentiation, a subtype typically characterized by heightened invasiveness and a higher propensity for hepatic metastasis (10). AFP-positive gastric cancer is recognized as a distinct subtype with unique biological behavior and clinical features, generally associated with poor prognosis. Early identification of such patients holds significant clinical importance (11). Carcinoembryonic antigen (CEA) is a highly glycosylated cell surface glycoprotein belonging to the immunoglobulin superfamily, whose expression is elevated in various epithelial-derived malignancies. The biological functions of CEA in gastric cancer include promoting tumor cell adhesion, inhibiting tumor cell apoptosis, and participating in cellular signal transduction (12). CEA serves not only as a critical adjunct in the diagnosis of gastric cancer but also plays a pivotal role in monitoring treatment response, predicting recurrence, and assessing prognosis. Studies showed that gastric cancer patients with elevated preoperative CEA levels had significantly lower survival rates compared to those with normal CEA levels (13).

CA125, AFP, and CEA are commonly used clinical tumor markers. Existing research (14) has applied the aforementioned factors to the diagnosis, treatment evaluation, and prognosis of digestive system tumors. However, these biomarkers often demonstrate inadequate specificity and sensitivity when used in isolation. CEA levels may be elevated in various digestive system tumors and certain non-neoplastic conditions, demonstrating limited diagnostic value when used in isolation. CA125 is more widely applied in ovarian cancer, whereas its specificity in gastric cancer diagnosis remains relatively underexplored. AFP is primarily utilized for hepatocellular carcinoma diagnosis but is also expressed in certain cases of gastric cancer, a pattern associated with specific molecular pathological alterations and prognostic implications, warranting further investigation.

This study presents a novel contribution by systematically evaluating, for the first time, the clinical utility of the combined detection of CA125, AFP, and CEA in the diagnosis and prognostic assessment of gastric cancer. Through ROC curve analysis, we quantitatively assessed the enhancement in diagnostic efficacy of combined detection compared to single-marker analysis. Furthermore, the correlation of these biomarkers with TNM staging, differentiation grade, pathological classification, and metastatic status were comprehensively investigated, thereby establishing a more integrative serological evaluation system for gastric cancer. Additionally, the prognostic value of these biomarkers was explored to provide a scientific basis for individualized clinical treatment strategies. Compared with previous studies, this research has a larger sample size, a longer follow-up period, more rigorous analytical methods, and greater clinically applicable conclusions. This study aimed to retrospectively analyze the diagnostic and prognostic value of CA125, CEA, and AFP individually and in combination for gastric cancer, thereby providing additional clinical insights for the diagnosis and treatment of gastric cancer.

## Materials and methods

### Study design and patients

This study was conducted with the approval of the ethics committee of Baoji People's Hospital [Approval No. (S001)-19] and utilized a retrospective cohort study design. The time period was set from January 2020 to November 2022, with a follow-up duration of 18 months (endpoint of May 2024). Patients who were treated in our hospital during the time period were screened according to the following inclusion and exclusion criteria:

Inclusion criteria for the gastric cancer group: (1) Pathologically confirmed diagnosis of gastric cancer; (2) Complete pathological examination results (TNM staging (14), degree of differentiation, gastric cancer classification); (3) Age ≥ 18 years; (4) Patients with complete baseline clinical data [gender, age, body mass index (BMI), underlying medical history] who underwent serological tests for CA125, AFP, and CEA at admission; (5) Definite clinical outcomes by the end of follow-up (May 2024) (tumor metastasis status, survival status); (6) Patients who received uniform clinical interventions, such as D2 radical gastrectomy for stage I–II patients, D2 radical gastrectomy followed by adjuvant chemotherapy with the XELOX regimen (oxaliplatin 130 mg/m^2^ via intravenous infusion on day 1 and capecitabine 1000 mg/m^2^ orally on days 1–14 of a 3-week cycle, for a total of 8 cycles) for stage III patients, and palliative surgery combined with systemic chemotherapy for stage IV patients. Exclusion criteria for the gastric cancer group: (1) Patients with recurrent gastric cancer; (2) Incomplete medical records; (3) Inadequate follow-up data; (4) Concurrent malignancies of other organs; (5) Pregnant or lactating women; (6) Immune system disorders; (7) Hematological diseases. After screening according to the above criteria, 98 patients with gastric cancer were ultimately selected as the gastric cancer group.

Inclusion criteria for the benign group: (1) Pathologically confirmed diagnosis of benign gastric lesions (leiomyoma, fibroma, neurofibroma); (2) Patients with complete baseline clinical data (gender, age, BMI, underlying medical history) who underwent serological tests for CA125, AFP, and CEA in the hospital; (3) Age ≥ 18 years. Exclusion criteria for the benign group: (1) Incomplete medical records; (2) Pregnant or lactating women; (3) Immune system disorders; (4) Hematological diseases. After screening according to the above criteria, 80 patients with gastric benign lesions were ultimately selected as the benign group.

### Data collection

The hospital's information system was used to collect the patient data in the gastric cancer group, including baseline clinical data (gender, age, BMI, underlying medical history), pathological results of lesions (TNM staging, differentiation status, lesion type), serological markers (CA125, AFP, CEA at admission), and follow-up information (metastasis status, death status). The baseline clinical data (gender, age, BMI, underlying medical history) and serological markers (CA125, AFP, CEA at admission) were collected from patients in the benign group.

### Observation indicators

(1) The differences in the levels of serological markers (CA125, AFP, CEA at admission) and their positivity rates [with critical values for CA125, AFP, and CEA of 35.00 U/ml, 8.78 ng/ml, and 5.00 ng/ml, respectively (15)] were compared between the gastric cancer group and the benign group. The diagnostic efficacy for gastric cancer of CA125, AFP, CEA, and their combined detection was calculated using ROC curve plotting (determining the optimal cutoff value based on the maximum Youden index). (2) Patients in the gastric cancer group were categorized based on distinct pathological features into stage I-II group and stage III-IV group, poorly differentiated group and moderately to well-differentiated group, and subgroups of papillary adenocarcinoma, signet ring cell carcinoma, and mucinous adenocarcinoma. The differences in CA125, AFP, and CEA among these subgroups were compared, and the diagnostic efficacy of CA125, AFP, CEA, and their combined detection for various pathological features of gastric cancer was calculated using ROC curve plotting (determining the optimal cutoff value based on the maximum Youden index). (3) According to the follow-up outcomes of 98 gastric cancer patients (endpoint of May 2024), they were classified into metastasis group and non-metastasis group, as well as death group and survival group. The differences in CA125, AFP, and CEA among patients with different prognoses were compared. The diagnostic efficacy of CA125, AFP, CEA, and their combined detection for different prognoses of gastric cancer was calculated using ROC curve plotting (determining the optimal cutoff value based on the maximum Youden index).

### Statistical analysis

The data entry for this study was conducted using Excel 2021, and data analysis was performed with SPSS 28.0. The measurement data (mean age, BMI, etc.) followed a normal distribution and were expressed as mean ± standard deviation. Inter-group differences were assessed using an independent sample t-test. Categorical data were expressed as rate, and inter-group differences were examined using a chi-square test. Diagnostic efficacy was analyzed by plotting ROC curves. To identify independent prognostic factors, a multivariable Cox proportional hazards regression model was employed, incorporating variables with a univariate analysis P-value < 0.1. Hazard ratios (HR) and 95% confidence intervals (CI) were calculated accordingly. P<0.05 indicates statistical significance.

## Results

### Comparison of baseline clinical data between the gastric cancer group and the benign group

The baseline clinical data, such as gender, age, BMI, and underlying medical history, were compared between the gastric cancer group and the benign group. The intergroup differences showed no statistical significance (P>0.05), indicating good comparability (**Table 1**).

**Table 1:** Comparison of baseline clinical data between the gastric cancer group and the benign group ( $\bar{x}\pm s$ )/[n (%)].

General clinical data		Gastric cancer group (n=98)	Benign group (n=80)	*t/χ^2^*	*P*
Gender	Male	56	50	0.645	0.422
	Female	43	30		
Mean age (years)		59.69±10.21	60.59±12.01	0.540	0.590
Mean BMI (kg/m^2^)		22.69±3.26	22.38±3.89	0.578	0.564
Underlying medical history	Hypertension	10	9	0.051	0.822
	Diabetes	8	4	0.701	0.402

### Comparison of CA125, AFP, CEA levels and their positive rates between the gastric cancer group and the benign group

**Figure 1:**
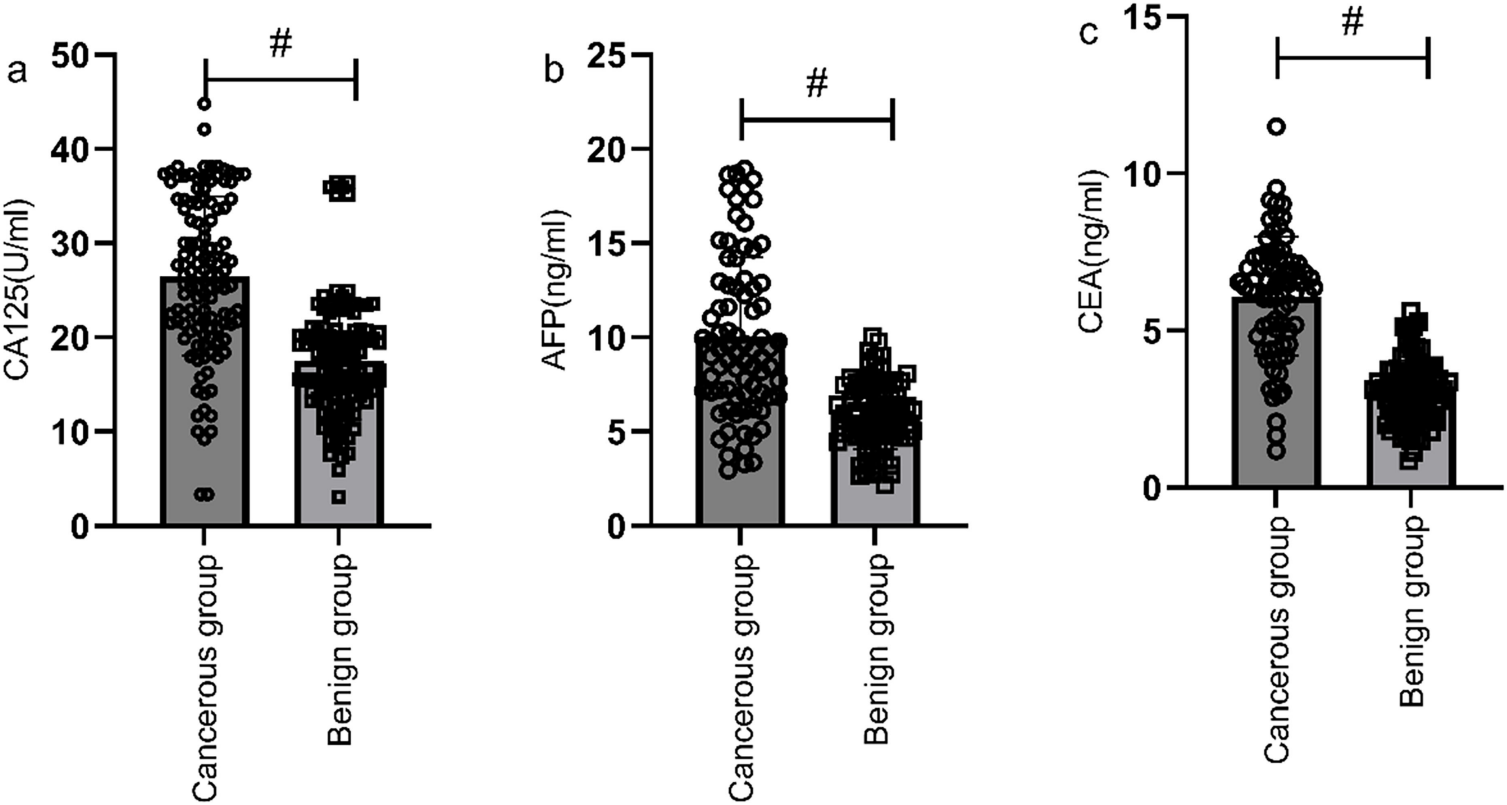
Comparison of CA125, AFP, and CEA levels between the gastric cancer group and the benign group. Serum levels of CA125 **(a)**, AFP **(b)**, and CEA **(c)** were significantly higher in the gastric cancer group compared to the benign group. # indicates a statistically significant difference for the same indicator between groups.

Serum levels of CA125, AFP, and CEA were significantly higher in the gastric cancer group compared to the benign group, with statistically significant difference (P<0.05) (**Figure 1**). The positive rates of serum CA125, AFP, and CEA in the gastric cancer group were also significantly higher compared to the benign group (6.98% vs. 1.51%, 4.16% vs. 1.01%, 19.86% vs. 5.98%), with statistically significant difference (P<0.05).

### Diagnostic value of serum CA125, AFP, and CEA in gastric cancer

**Figure 2:**
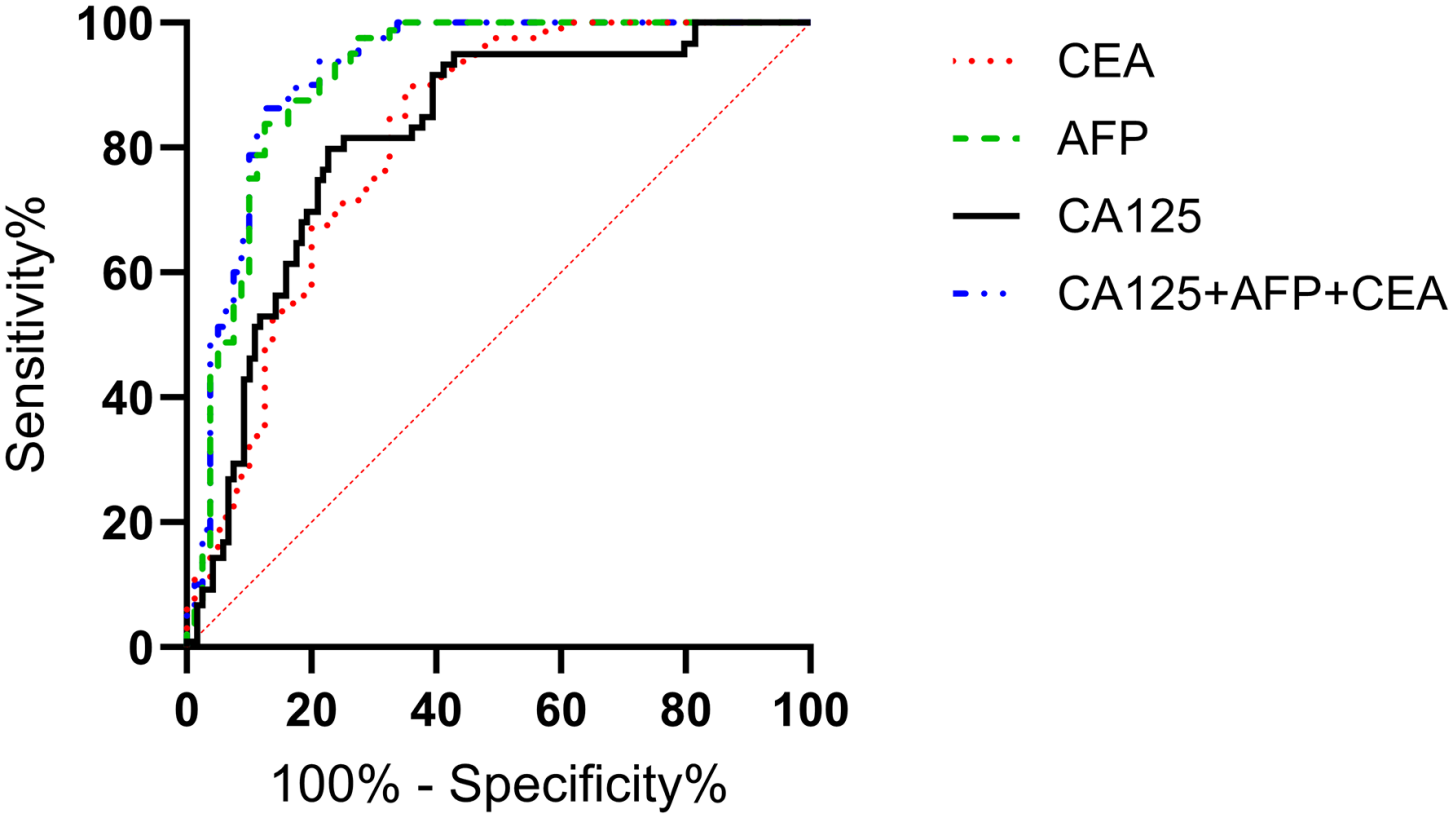
Analysis of the diagnostic value of serum CA125, AFP, and CEA in gastric cancer. The AUCs for CA125, AFP, CEA, and their combined detection in diagnosing gastric cancer were 0.815, 0.813, 0.911, and 0.919, respectively (P<0.001).

The ROC curves of serum CA125, AFP, CEA, and their combined detection for diagnosing gastric cancer were respectively plotted, and the AUCs were 0.815 (95% CI=0.758-0.871, P<0.001), 0.813 (95% CI=0.746-0.880, P<0.001), 0.911 (95% CI=0.863-0.959, P<0.001), and 0.919 (95% CI=0.873-0.965, P<0.001), respectively (**Table 2**, **Figure 2**).

**Table 2:** Diagnostic value of serum CA125, AFP, and CEA in gastric cancer.

Diagnostic indicator	AUC	SE	95% CI	P
CA125	0.815	0.029	0.758-0.871	<0.001
AFP	0.813	0.034	0.746-0.880	<0.001
CEA	0.911	0.025	0.863-0.959	<0.001
Combined detection	0.919	0.023	0.873-0.965	<0.001

### Correlation analysis of pathological features with serological markers in patients with gastric cancer

#### Correlation analysis of clinical staging and serological markers

**Figure 3:**
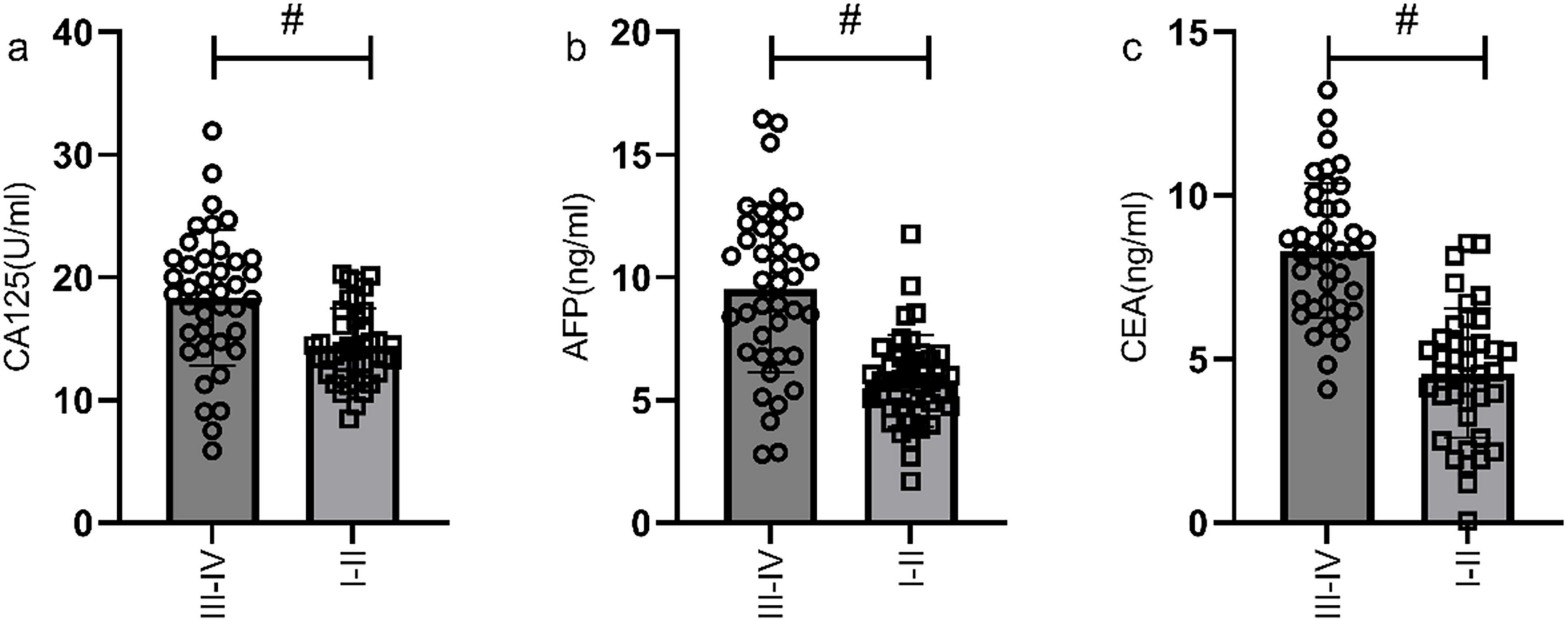
Comparison of serum CA125, AFP, and CEA levels in gastric cancer patients with different TNM stages. The serum levels of CA125 **(a)**, AFP **(b)**, and CEA **(c)** in stage III-IV patients were significantly higher than those in stage I-II patients. # indicates a statistically significant difference for the same indicator between groups.

**Figure 4:**
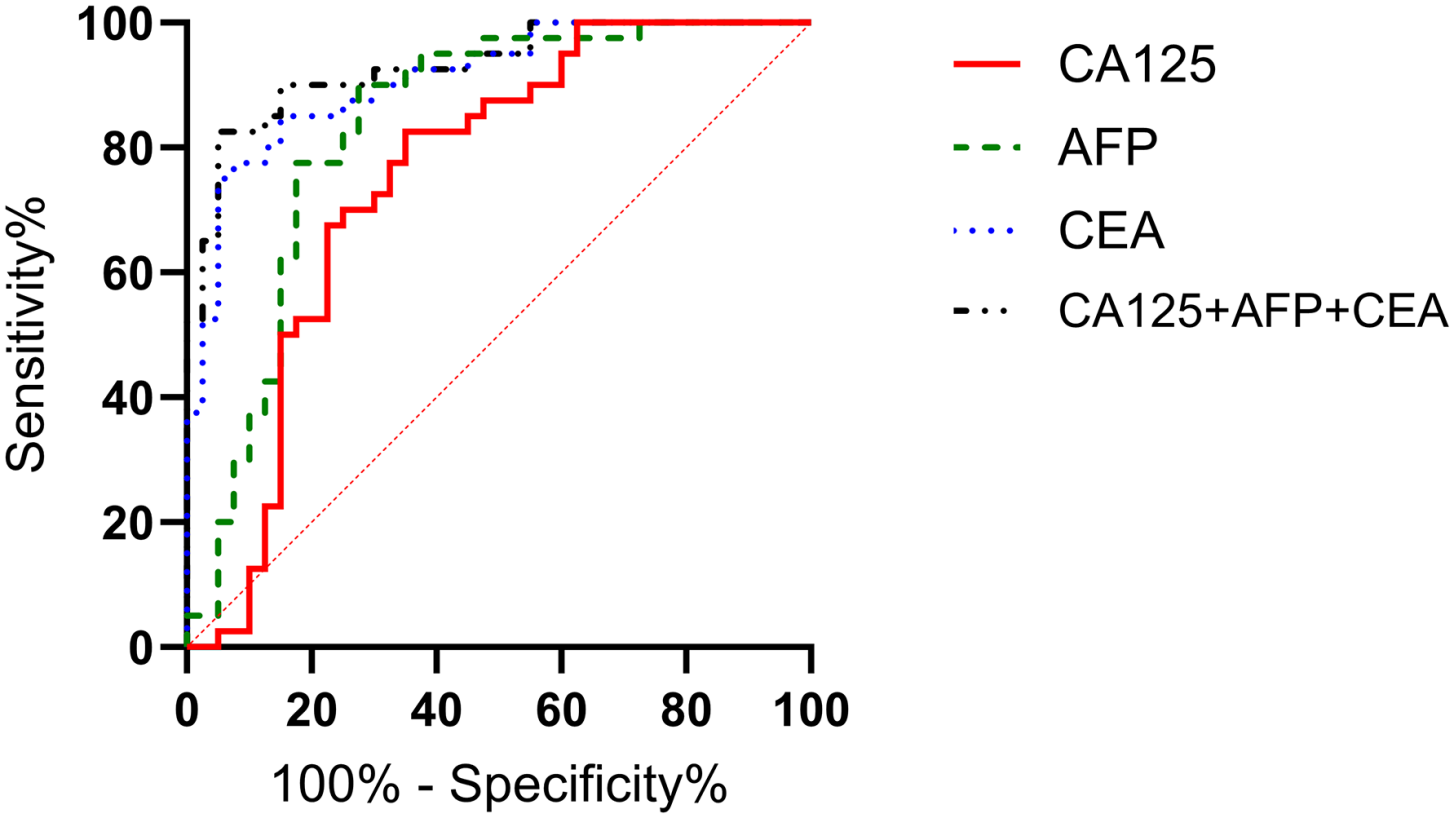
Diagnostic value of serum CA125, AFP, and CEA in different stages of gastric cancer. The AUCs for serum CA125, AFP, CEA, and their combined detection in TNM staging of gastric cancer were 0.751, 0.834, 0.911, and 0.931, respectively (P<0.001).

According to the 7th edition of the TNM classification by the International Union Against Cancer, 98 gastric cancer patients were divided into stage I-II group (n=60) and stage III-IV group (n=38). The serum levels of CA125, AFP, and CEA in stage III-IV patients were significantly higher than those in stage I-II patients (P<0.05), as shown in **Figure 3**. The ROC curves of serum CA125, AFP, CEA, and their combined detection for the TNM staging of gastric cancer were respectively plotted, and the AUCs were 0.751, 0.834, 0.911, and 0.931, respectively (P<0.001) (**Table 3**, **Figure 4**).

**Table 3:** Diagnostic value of serum CA125, AFP, and CEA in different stages of gastric cancer.

Diagnostic indicator	AUC	SE	95% CI	P
CA125	0.751	0.057	0.639-0.863	0.000
AFP	0.834	0.481	0.740-0.928	<0.001
CEA	0.911	0.031	0.850-0.973	<0.001
Combined detection	0.931	0.027	0.878-0.985	<0.001

### Correlation analysis of differentiation degree and serological markers

**Figure 5:**
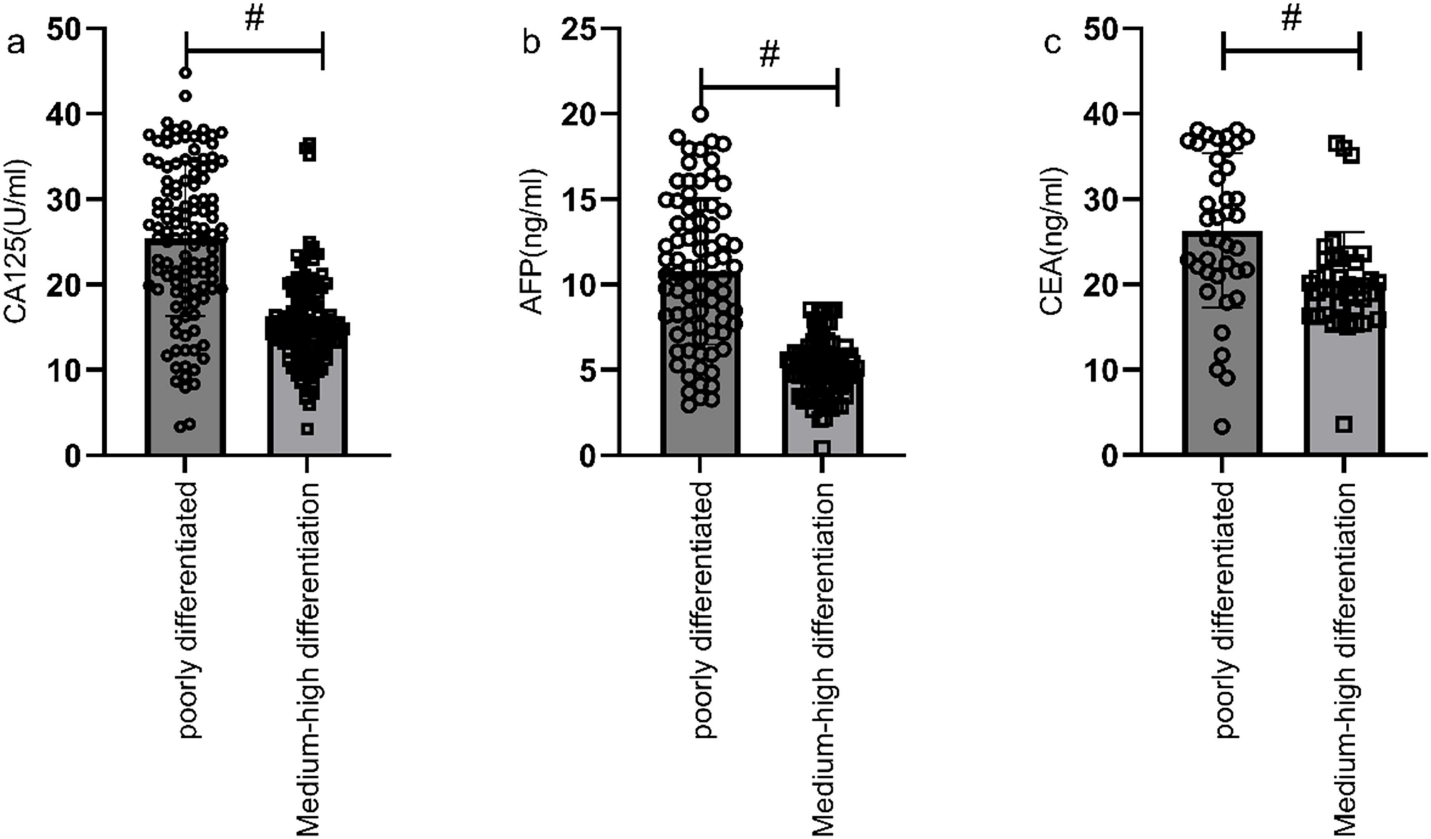
Comparison of serological markers in gastric cancer patients with different differentiation degrees. Patients in poorly differentiated group had higher levels of CA125 **(a)**, AFP **(b)**, and CEA **(c)** compared to those in moderately to well-differentiated group (P<0.05). # indicates a statistically significant difference for the same indicator between groups.

**Figure 6:**
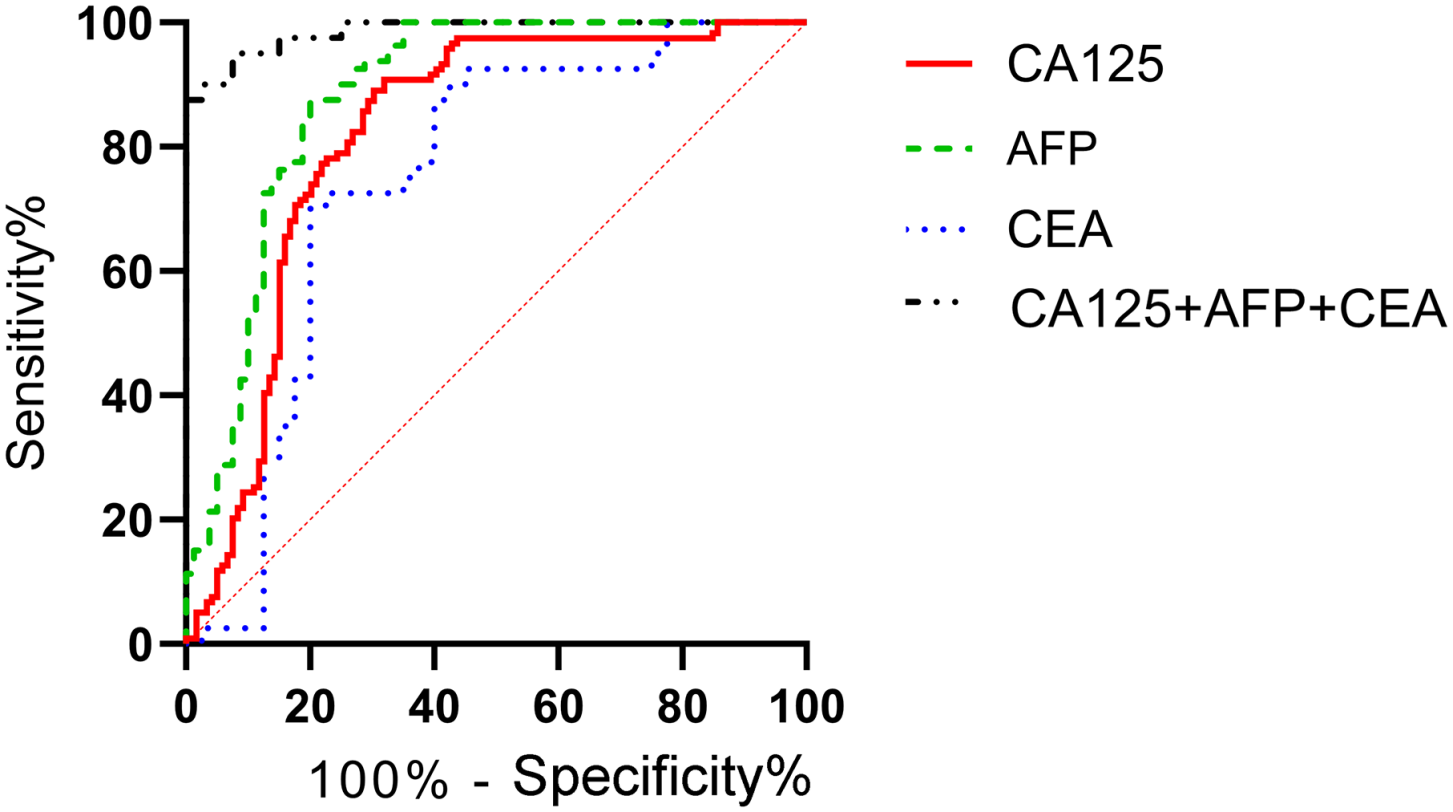
Diagnostic value of serum CA125, AFP, and CEA in different differentiation degrees of gastric cancer. The ROC curve of serum CA125, AFP, CEA, and their combined detection for different differentiation degrees of gastric cancer showed that the AUCs were 0.819, 0.883, 0.746, and 0.986, respectively (P<0.001).

According to the patients' postoperative pathological findings, 98 patients with gastric cancer were differentiated into a poorly differentiated group (n=39) and a moderately to well-differentiated group (n=59). Patients in poorly differentiated group had higher levels of CA125, AFP, and CEA compared to those in moderately to well-differentiated group (P<0.05) (**Figure 5**). Furthermore, by plotting the ROC curve, the calculated AUCs for serum CA125, AFP, CEA, and their combined detection in diagnosing the differentiation degree of gastric cancer were 0.819, 0.883, 0.746, and 0.986 (P<0.001), respectively, as shown in **Table 4** and **Figure 6**.

**Table 4:** Diagnostic value of serum CA125, AFP, and CEA in different differentiation degrees of gastric cancer.

Diagnostic indicators	AUC	SE	95% CI	P
CA125	0.819	0.029	0.762-0.877	<0.001
AFP	0.883	0.028	0.828-0.938	<0.001
CEA	0.746	0.059	0.632-0.861	<0.001
Combined detection	0.986	0.009	0.968-1.000	<0.001

### Correlation analysis of different gastric cancer types and serological markers

**Figure 7:**
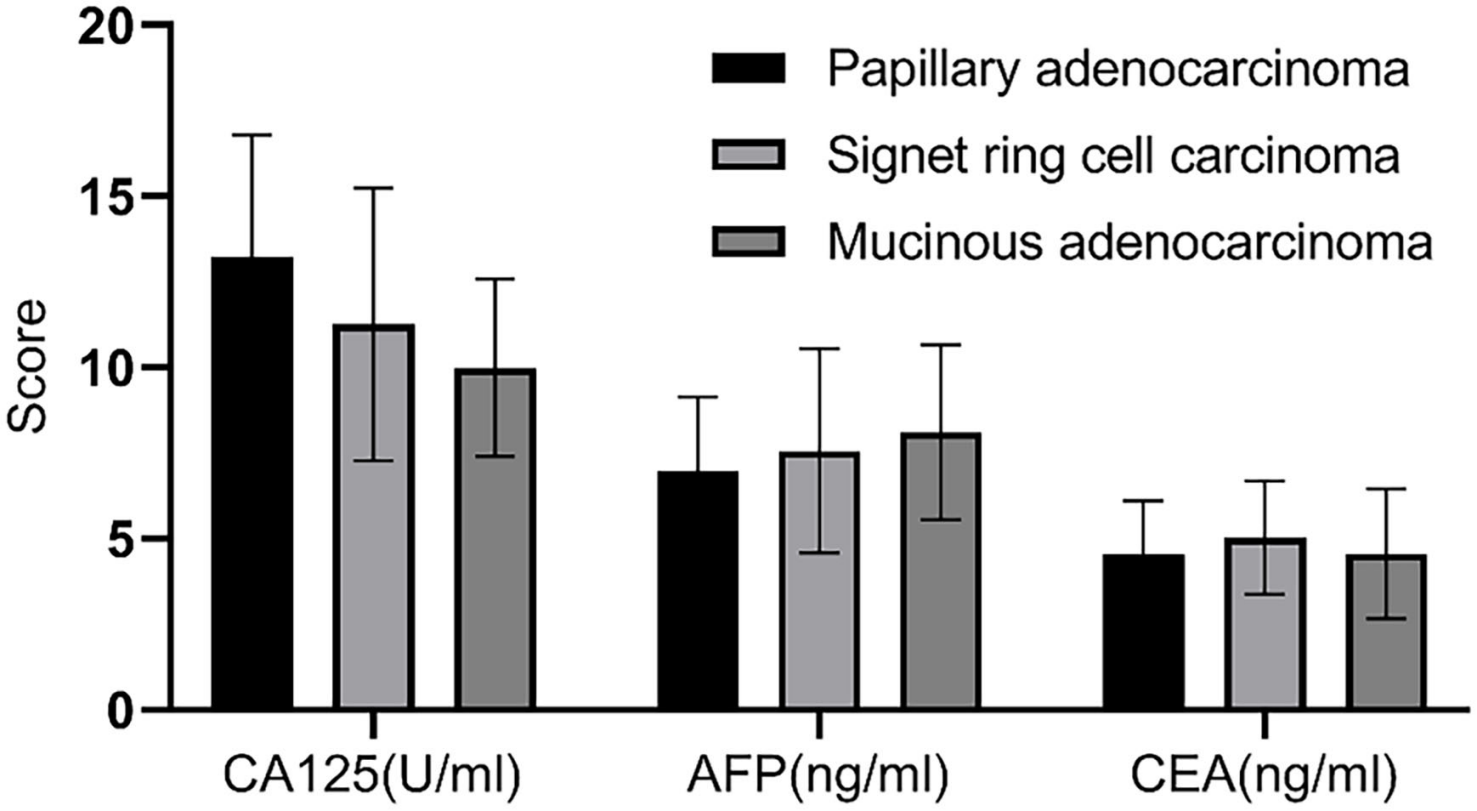
Correlation analysis of different gastric cancer types and serological markers. There was no statistically significant difference in the serum levels of CA125, AFP, and CEA among patients with different types of gastric cancer (P>0.05).

Based on pathological results, 98 gastric cancer patients were classified into papillary adenocarcinoma (n=77), signet ring cell carcinoma (n=14), and mucinous adenocarcinoma (n=7). Comparison revealed no statistically significant differences in serum levels of CA125, AFP, and CEA among the different types of gastric cancer patients (P>0.05), as shown in **Figure 7**.

### Correlation analysis of prognosis and serological markers in gastric cancer patients

#### Correlation analysis of metastasis status and serological markers

**Figure 8:**
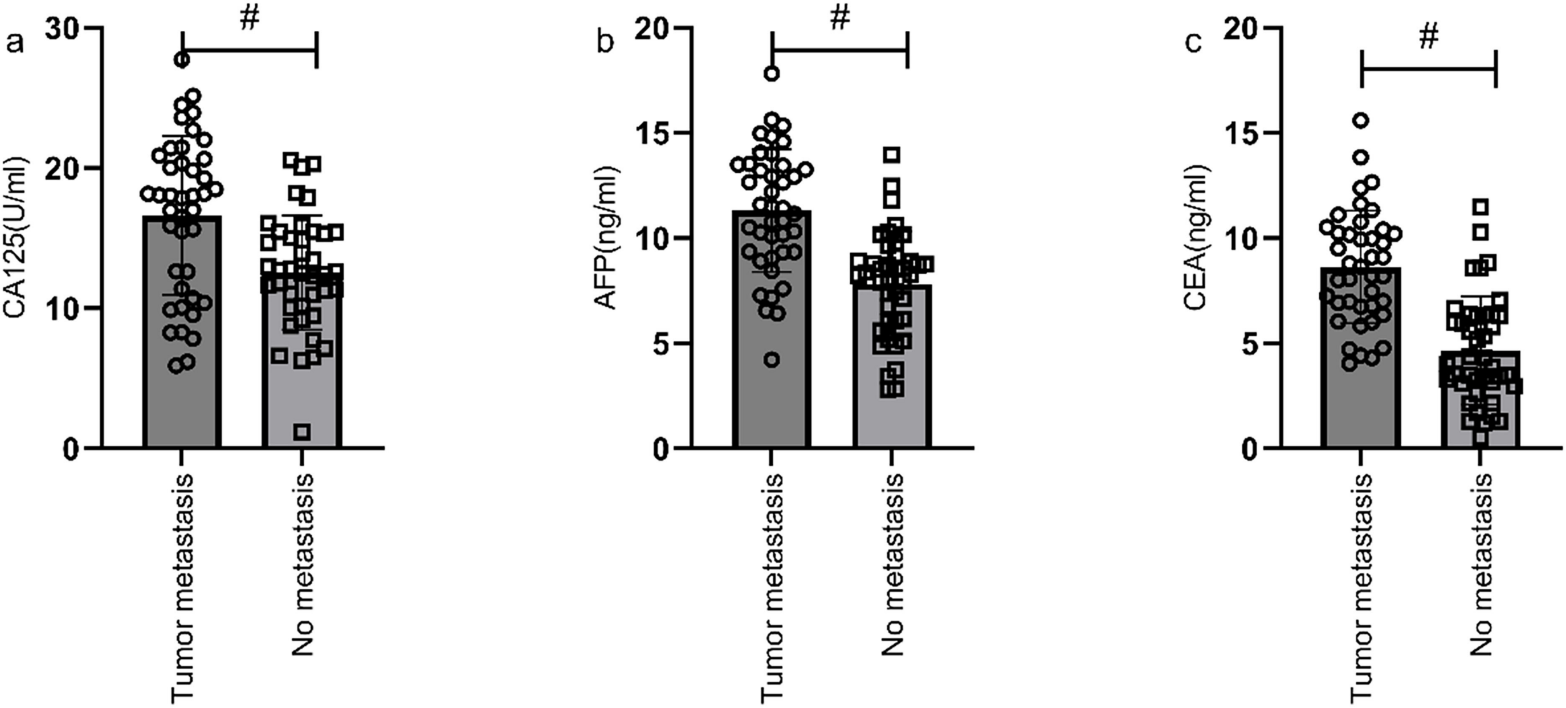
Correlation analysis of metastasis status and serological markers. Patients in metastasis group had higher levels of CA125 **(a)**, AFP **(b)**, and CEA **(c)** compared to those in non-metastasis group (P<0.05). # indicates a statistically significant difference for the same indicator between groups.

**Figure 9:**
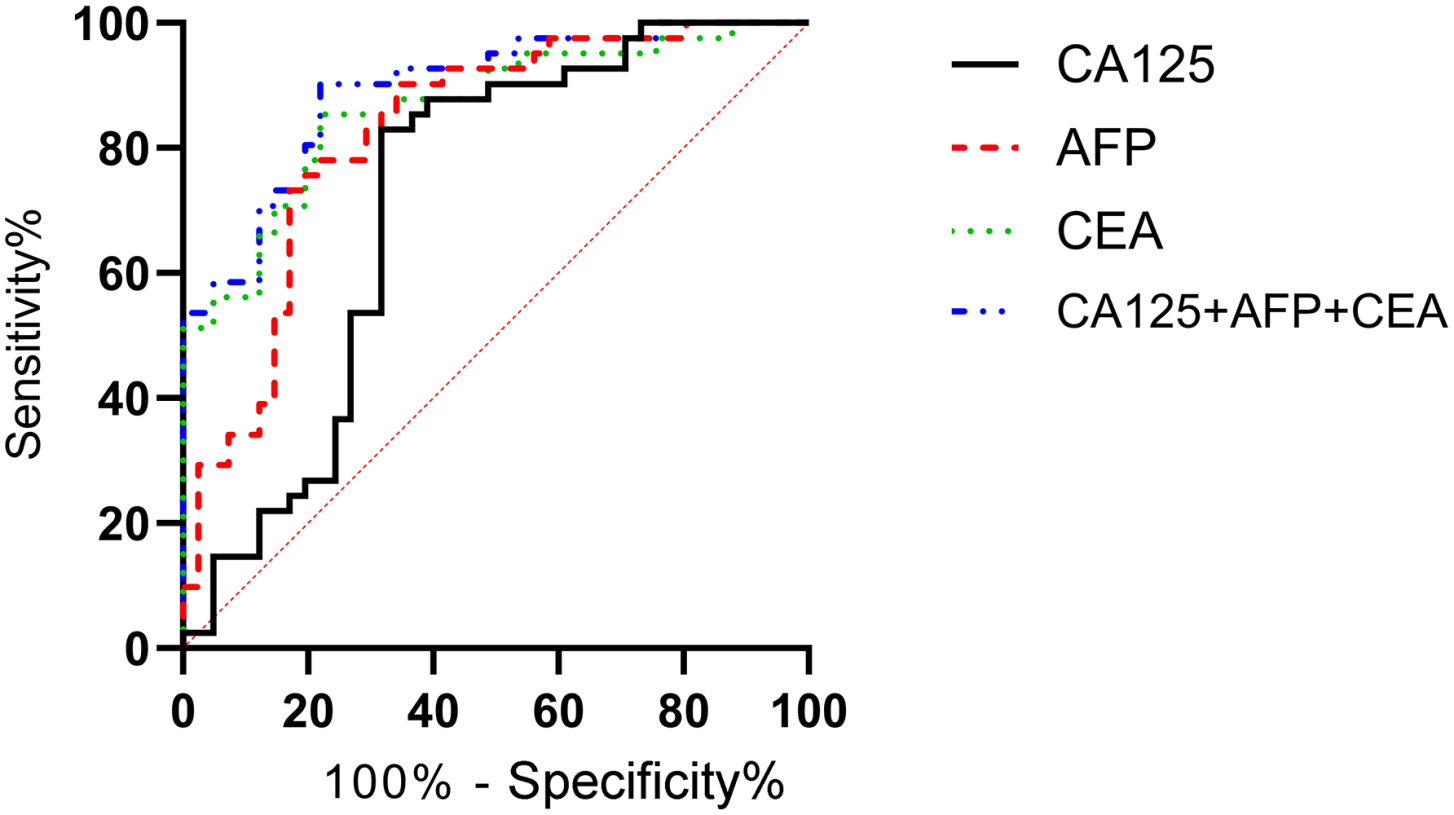
Diagnostic value of serum CA125, AFP, and CEA in metastasis status of gastric cancer. The AUCs for serum CA125, AFP, CEA, and their combined detection in diagnosing metastasis were 0.716, 0.825, 0.863, and 0.892, respectively (P<0.001).

According to the follow-up outcomes of 98 gastric cancer patients, they were classified into metastasis group (n=41) and non-metastasis group (n=57). Patients with metastasis had higher levels of CA125, AFP, and CEA compared to those without metastasis (P<0.05) (**Figure 8**). Further analysis through ROC curve plotting revealed that the AUCs for serum CA125, AFP, CEA, and their combined detection in diagnosing metastasis of gastric cancer were 0.716, 0.825, 0.863, and 0.892, respectively (P<0.001) (**Table 5**, **Figure 9**).

**Table 5:** Diagnostic value of serum CA125, AFP, and CEA in metastasis status of gastric cancer.

Diagnostic indicators	AUC	SE	95% CI	P
CA125	0.716	0.060	0.600-0.833	<0.001
AFP	0.825	0.047	0.733-0.916	<0.001
CEA	0.863	0.040	0.784-0.942	<0.001
Combined detection	0.892	0.035	0.824-0.960	<0.001

### Correlation analysis of survival status and serological markers

**Figure 10:**
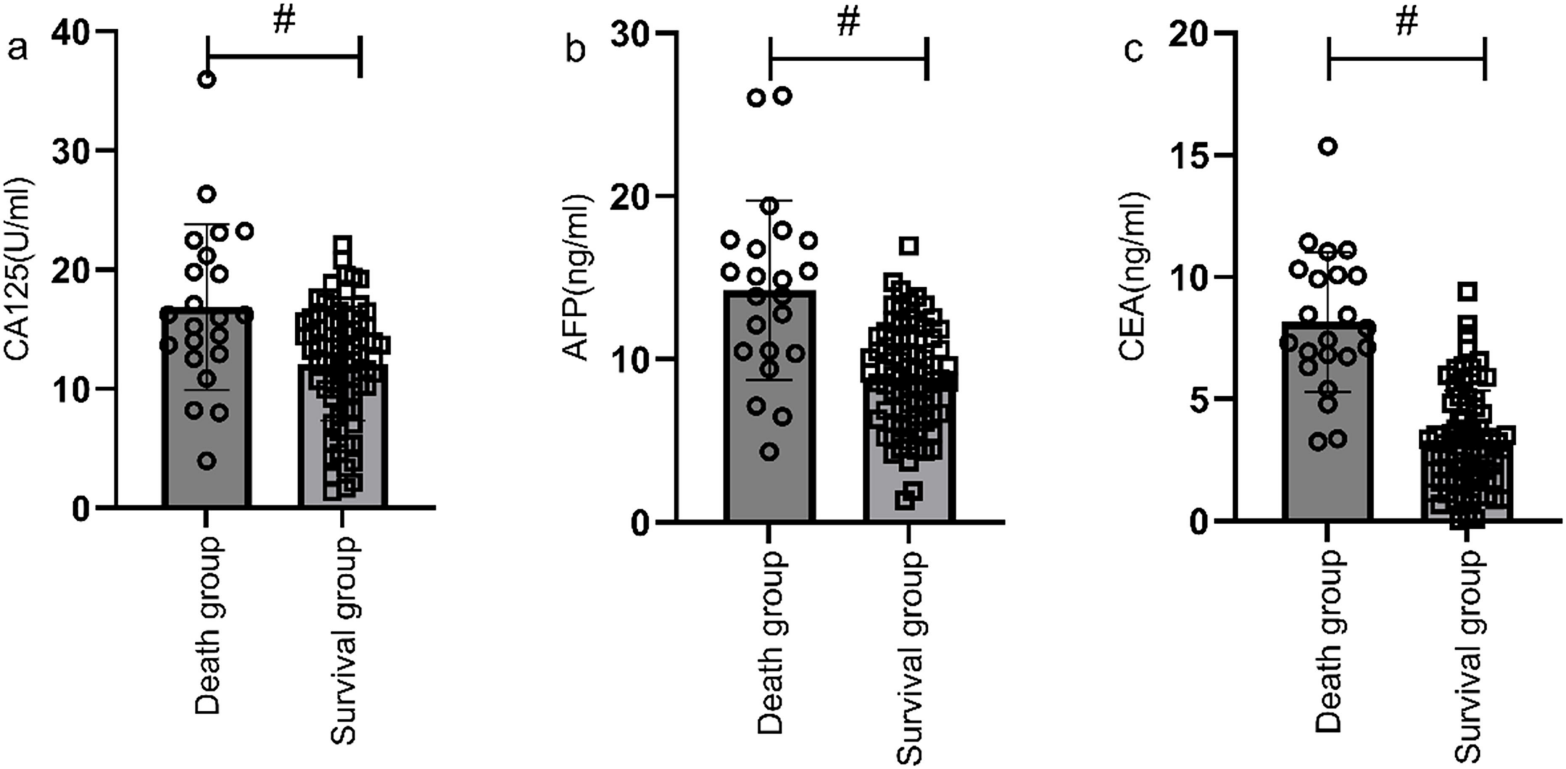
Correlation analysis of survival status and serological markers. The levels of CA125 **(a)**, AFP **(b)**, and CEA **(c)** of patients in the death group were higher than those in the survival group (P<0.05). # indicates a statistically significant difference for the same indicator between groups.

**Figure 11:**
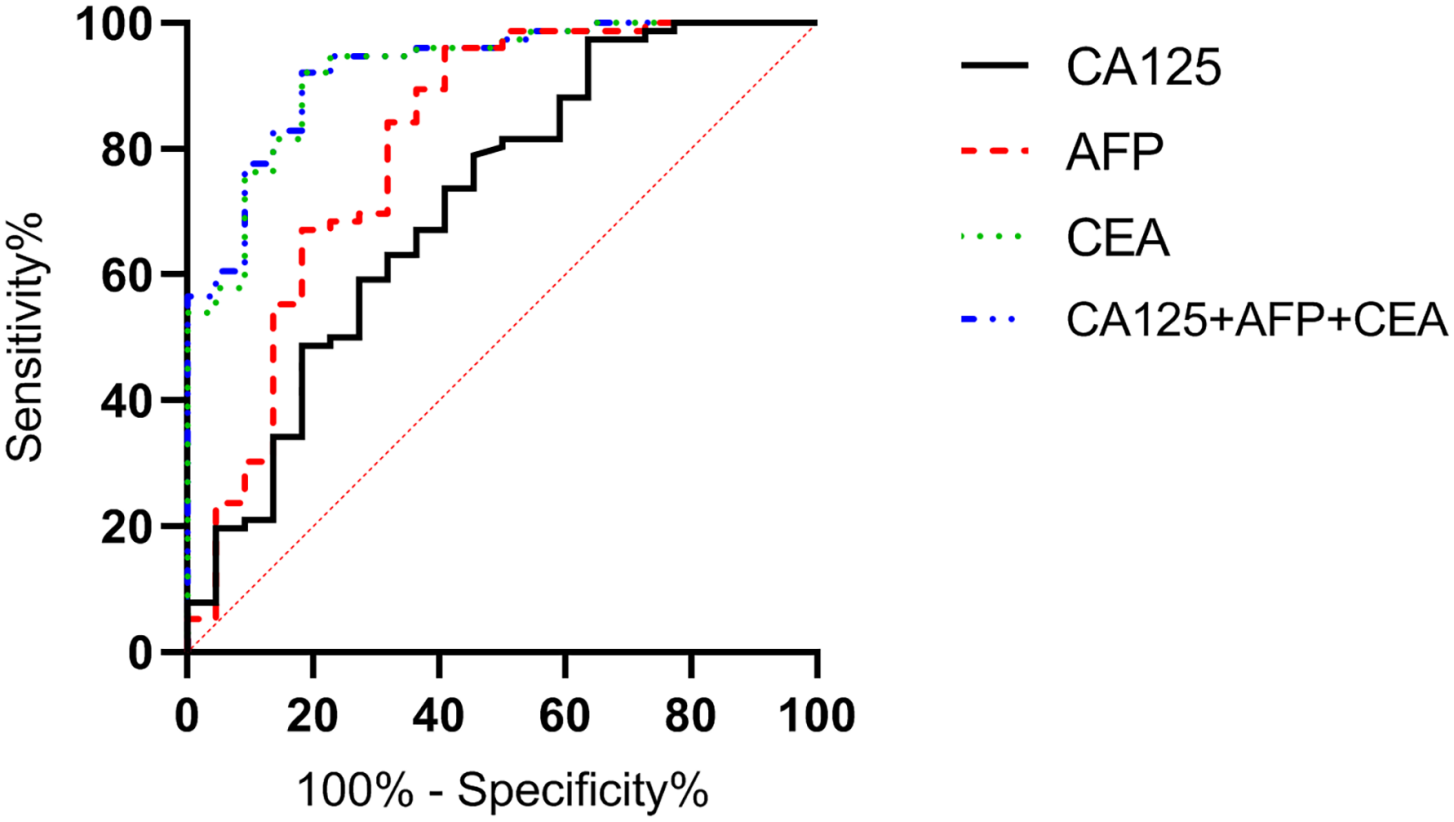
Diagnostic value of serum CA125, AFP, and CEA in clinical outcomes of gastric cancer patients. The AUCs for serum CA125, AFP, CEA, and their combined detection in predicting clinical outcomes of gastric cancer patients were 0.713, 0.809, 0.922, and 0.926, respectively (P<0.001).

According to the follow-up outcomes of 98 gastric cancer patients, they were classified into death group (n=22) and survival group (n=76). The levels of CA125, AFP, and CEA of patients in the death group were higher than those in the survival group (P<0.05) (**Figure 10**). The AUCs for serum CA125, AFP, CEA, and their combined detection in predicting clinical outcomes of gastric cancer patients were 0.713, 0.809, 0.922, and 0.926, respectively (P<0.001) (**Table 6**, **Figure 11**).

**Table 6:** Diagnostic value of serum CA125, AFP, and CEA in clinical outcomes of gastric cancer patients.

Diagnostic indicators	AUC	SE	95% CI	P
CA125	0.713	0.067	0.582-0.844	0.002
AFP	0.809	0.062	0.688-0.930	<0.001
CEA	0.922	0.031	0.861-0.984	<0.001
Combined detection	0.926	0.030	0.867-0.985	<0.001

### Multivariable prognostic analysis of gastric cancer

Using patient survival status during follow-up as the dependent variable and other factors (such as sex, peritoneal metastasis, and TNM staging) as independent variables, both univariate and multivariable Cox regression analyses were conducted. Univariate analysis revealed that TNM staging, degree of differentiation, peritoneal metastasis, and the serum levels of CA125, AFP, and CEA were significantly associated with patient survival (P<0.05) (**Table 7**). Further multivariable analysis identified TNM staging, peritoneal metastasis, and CEA level as independent prognostic factors (P<0.05) (**Table 8**).

**Table 7:** Univariate Cox regression analysis of mortality in patients with gastric cancer.

Variable	B	S.E	Wald	95% CI	P
Male/Female	0.123	0.326	0.155	1.13 (0.61-2.06)	0.639
Age (≥60 years or not)	0.296	0.306	0.996	1.34 (0.76-2.43)	0.333
TNM staging (I-II vs III-IV)	0.886	0.202	19.325	2.412 (1.63-3.65)	<0.001
Degree of differentiation (poorly differentiated vs moderately to well-differentiated)	0.631	0.216	8.635	1.86 (1.21-2.68)	0.005
Gastric cancer types	0.619	0.265	0.469	1.19 (0.76-1.96)	0.463
Peritoneal metastasis	1.132	0.216	28.516	2.89 (2.06-4.69)	<0.001
CA125 (High vs Normal)	0.516	0.231	5.063	1.69 (1.05-2.65)	0.023
AFP (High vs Normal)	0.556	0.236	5.896	1.75 (1.12-2.69)	0.015
CEA (High vs Normal)	0.715	0.226	11.236	2.32 (1.36-3.26)	0.009

**Table 8:** Multivariable Cox regression analysis of mortality in patients with gastric cancer.

Variable	B	S.E	Wald	95% CI	P
TNM staging (I-II vs III-IV)	0.635	0.229	8.562	1.92 (1.21-2.98)	0.003
Peritoneal metastasis	0.936	0.227	16.325	2.51 (1.63-3.69)	<0.001
CEA (High vs Normal)	0.216	0.269	1.156	1.32 (0.79-2.26)	0.023

## Discussion

Gastric cancer refers to a malignant tumor originating from the epithelium of the gastric mucosa, which is a prevalent disease in the field of gastroenterology and one of the most common tumors worldwide (16). Epidemiological studies in China show that the mortality rate of gastric cancer has risen from the fourth place in the 1970s to the foremost position, and all these data suggest that gastric cancer has posed a serious threat to the life and health of the residents (17). Early clinical manifestations of gastric cancer are often subtle, resembling benign conditions such as gastritis, thus failing to elicit adequate attention from patients, and once detected, the disease has frequently progressed to the advanced stage; the clinical symptoms in advanced gastric cancer patients include hematemesis, severe pain, etc., significantly compromising the quality of life of patients, especially the elderly patients, so early diagnosis, timely intervention, and close assessment are pivotal factors influencing the prognosis of gastric cancer patients (18, 19).

This study utilized the clinically prevalent tumor markers CA125, AFP, and CEA as research indicators to analyze their respective application value in gastric cancer patients with various pathological features and prognoses. The results suggested that the serum levels of CA125, AFP, and CEA were significantly higher in patients with gastric cancer than in those with benign lesions. This conclusion has been corroborated by numerous studies. For instance, Tong et al. (20) indicated that AFP, CEA, CA199, CA125, and CA724 were commonly used clinical serum tumor markers, which played a crucial role in the diagnosis, prognosis, and recurrence monitoring of gastrointestinal malignancies, and that they identified CA125 as an independent prognostic risk factor for gastric cancer patients through ROC curve plotting and Cox regression analysis. However, unlike the findings of Tong et al., our study revealed that CA125 was not only a prognostic factor but also closely associated with the differentiation of gastric cancer, with an AUC value reaching 0.819. This finding fills in the lack of previous research regarding the correlation between CA125 and gastric cancer differentiation. This correlation may stem from the heightened epithelial-mesenchymal transition (EMT) capacity of poorly differentiated gastric cancer cells, with CA125, an epithelial-derived tumor marker, potentially directly involving in this process. Although this hypothesis requires further validation through fundamental experiments, it has already provided a novel perspective for utilizing CA125 in assessing the differentiation of gastric cancer in clinical practice. CEA is a structurally complex and highly diverse soluble glycoprotein formed in the cytoplasm, often adhering to the surface of tumor cells, and it functions to block tumor cell apoptosis and signal transduction, making it a broad-spectrum tumor marker. CA125 is predominantly present in the epithelial cells of the respiratory and digestive tracts, as well as the reproductive organs, and it promotes tumor cell growth and prevents apoptosis. AFP is frequently produced by the fetal liver and yolk sac and is a common marker for gastrointestinal tumors, gallbladder cancer, and lung cancer. The results of this study indicated that the serum levels of CA125, AFP, and CEA in gastric cancer patients were significantly elevated, suggesting that these markers may have diagnostic efficacy for gastric cancer. Through an in-depth analysis of the biological significance of these three biomarkers, it was found that they may reflect distinct aspects of gastric cancer progression. Elevated CEA is likely associated with uncontrolled tumor cell proliferation and resistance to apoptosis. Increased CA125 may predominantly indicate alterations in the tumor microenvironment, particularly the likelihood of peritoneal metastasis. Meanwhile, elevated AFP may suggest fetal-like tumor characteristics, potentially linked to hepatic differentiation or stem cell-like properties. This multifaceted reflection significantly enhances the diagnostic value of combined detection, far surpassing that of individual biomarkers. This also explains why the combined detection in our study achieved an AUC of 0.919 for gastric cancer diagnosis, markedly outperforming individual biomarkers.

The study further subdivided the gastric cancer patients according to different pathological features, revealing that CA125, AFP, and CEA levels in stage III-IV patients were higher than in stage I-II patients, and that poorly differentiated patients exhibited higher CA125, AFP, and CEA levels than moderately to well-differentiated patients. These results suggest a correlation between serological markers and the pathological features of gastric cancer patients. This has also been confirmed by other scholars. For instance, Ueda et al. (21) found that serum CA125 levels in advanced gastric cancer patients with peritoneal metastasis were significantly higher than in those without peritoneal metastasis, and they suggested that CA125 could be used as a clinical indicator to assess the risk of peritoneal metastasis during the treatment of advanced gastric cancer. Although both Wang et al. (22) and our study confirmed the correlation between serum biomarkers and gastric cancer staging, it was noteworthy that our research further revealed distinct expression patterns of these three biomarkers in different stages of gastric cancer. Specifically, CEA exhibited significantly superior diagnostic efficacy for stage III-IV gastric cancer (AUC = 0.911) compared to CA125 (AUC = 0.751). This presents an intriguing contrast to the prevailing notion that CA125 is more sensitive to peritoneal metastasis. This discrepancy may reflect the characteristics of our sample, wherein advanced gastric cancer patients predominantly exhibited hematogenous metastasis and local infiltration rather than peritoneal dissemination. These findings underscore the importance of individualized assessment, as gastric cancer patients with different metastatic patterns may require attention to distinct serum biomarkers. In this study, ROC curve analysis was conducted to calculate the correlation of individual indicators and combined detection with pathological features of gastric cancer, leading to two conclusions: first, CEA, CA125, and CA199 are correlated with the pathological features of gastric cancer patients; second, combined detection has superior diagnostic efficacy compared to individual indicators (23). These conclusions can be applied in the initial screening of gastric cancer, providing positive implications for reducing medical costs associated with initial screening.

In this study, patients were also divided into subgroups based on their follow-up outcomes, suggesting that metastasis and death statuses are correlated with CEA, CA125, and CA199 levels in gastric cancer patients, and that combined detection achieved an AUC of 0.892 for diagnosing metastasis status and 0.926 for diagnosing death status. Similar findings have been observed in other studies. For instance, research by Yang et al. (24) indicated that patients with high CA125 and CEA expression had a shorter median survival time compared to those with low CA125 and CEA expression. Zhang et al. (25) also indicated that using Kaplan-Meier curves and multivariate Cox proportional models, CEA and AFP were independent prognostic factors for gastric cancer patients, recommending more rigorous follow-up measures for those with high levels of these indicators. Tumor markers are often closely associated with the growth and metastasis of malignant tumors (26). Abnormally elevated levels of these markers typically indicate higher tumor aggressiveness, leading to poorer patient prognosis. This information is significant for guiding the treatment of patients with malignant tumors.

Finally, the study further employed multivariable Cox regression analysis to investigate the independent associations between tumor markers and overall survival in gastric cancer patients. The results indicated that TNM staging, peritoneal metastasis, and CEA levels were significant prognostic factors for gastric cancer, whereas the independent associations of CA125 and AFP with survival were attenuated. We posit that the weak association of CA125 and AFP with the prognosis of gastric cancer patients may stem from their roles as indicators of peritoneal metastasis rather than as direct causal factors. In contrast, the strong association of CEA with prognosis is likely attributable to its direct involvement in tumor progression rather than serving merely as a marker of disease progression.

The findings indicated an association between serum biomarkers and gastric cancer prognosis; however, it is essential to recognize that the prognosis of gastric cancer is influenced by a multitude of factors. Clinically, patient outcomes are determined not only by the biological characteristics of the tumor but also by variables such as the surgical approach (D1/D2 radical gastrectomy), adjuvant treatment strategies (neoadjuvant chemotherapy or concurrent chemoradiotherapy), and postoperative surveillance protocols. In this study, although basic treatment regimens were controlled (patients received uniform clinical interventions), there remain certain limitations in relying solely on tumor markers for prognostic assessment. In clinical practice, serum biomarkers such as CA125, AFP, and CEA should be regarded as adjunctive tools in prognostic assessment, rather than definitive indicators, necessitating a comprehensive evaluation that integrates pathological features and therapeutic strategies.

## Conclusion

Serum levels of CA125, AFP, and CEA in patients with gastric cancer were significantly elevated and were correlated with the degree of differentiation and TNM staging. Combined detection had diagnostic efficacy in assessing metastasis and clinical outcomes, providing valuable guidance for clinical treatment of patients with gastric cancer. This study is innovative in two aspects: first, it validated the diagnostic value of the combined detection of CA125, AFP, and CEA for gastric cancer; second, it confirmed the close association of CA125, AFP, and CEA with the prognosis of gastric cancer patients. These findings offer valuable guidance for the clinical treatment and follow-up of gastric cancer patients. The limitations of this study lie in its retrospective design, single-center nature, and relatively small sample size. Furthermore, despite controlling for basic therapeutic regimens, we were unable to perform a detailed analysis of the prognostic impact of interactions between different surgical approaches (e.g., D1/D2 radical surgery) and specific adjuvant treatments in relation to serum biomarkers. Future studies should expand the sample size, conduct multicenter prospective investigations, and explore the development of an integrated prognostic scoring system that incorporates serum biomarkers, pathological features, and therapeutic modalities, to enhance the accuracy of prognostic assessment in gastric cancer patients.

## Data Availability

The original contributions presented in the study are included in the article/supplementary material. Further inquiries can be directed to the corresponding author.
